# Retinal nerve fibre layer thinning is associated with drug resistance in epilepsy

**DOI:** 10.1136/jnnp-2015-310521

**Published:** 2015-04-17

**Authors:** Simona Balestrini, Lisa M S Clayton, Ana P Bartmann, Krishna Chinthapalli, Jan Novy, Antonietta Coppola, Britta Wandschneider, William M Stern, James Acheson, Gail S Bell, Josemir W Sander, Sanjay M Sisodiya

**Affiliations:** 1 Department of Clinical and Experimental Epilepsy, NIHR University College London Hospitals Biomedical Research Centre, UCL Institute of Neurology, London, UK; 2 Neuroscience Department, Polytechnic University of Marche, Ancona, Italy; 3 Department of Neuro-Ophthalmology, National Hospital for Neurology and Neurosurgery, London, UK; 4 Stichting Epilepsie Instellingen Nederland, Heemstede (SEIN), Heemstede, The Netherlands

## Abstract

**Objective:**

Retinal nerve fibre layer (RNFL) thickness is related to the axonal anterior visual pathway and is considered a marker of overall white matter ‘integrity’. We hypothesised that RNFL changes would occur in people with epilepsy, independently of vigabatrin exposure, and be related to clinical characteristics of epilepsy.

**Methods:**

Three hundred people with epilepsy attending specialist clinics and 90 healthy controls were included in this cross-sectional cohort study. RNFL imaging was performed using spectral-domain optical coherence tomography (OCT). Drug resistance was defined as failure of adequate trials of two antiepileptic drugs to achieve sustained seizure freedom.

**Results:**

The average RNFL thickness and the thickness of each of the 90° quadrants were significantly thinner in people with epilepsy than healthy controls (p<0.001, t test). In a multivariate logistic regression model, drug resistance was the only significant predictor of abnormal RNFL thinning (OR=2.09, 95% CI 1.09 to 4.01, p=0.03). Duration of epilepsy (coefficient −0.16, p=0.004) and presence of intellectual disability (coefficient −4.0, p=0.044) also showed a significant relationship with RNFL thinning in a multivariate linear regression model.

**Conclusions:**

Our results suggest that people with epilepsy with no previous exposure to vigabatrin have a significantly thinner RNFL than healthy participants. Drug resistance emerged as a significant independent predictor of RNFL borderline attenuation or abnormal thinning in a logistic regression model. As this is easily assessed by OCT, RNFL thickness might be used to better understand the mechanisms underlying drug resistance, and possibly severity. Longitudinal studies are needed to confirm our findings.

## Introduction

The retinal nerve fibre layer (RNFL) contains glia and the unmyelinated axons of retinal ganglion cells. As retinal axons are devoid of myelin until they penetrate the lamina cribrosa, the evaluation of RNFL thickness has been suggested as a method of assessing axonal ‘integrity’ in the anterior visual pathway and the white matter tracts throughout the central nervous system.^1^ Optical coherence tomography (OCT) is a reproducible and non-invasive technique for cross-sectional imaging of retinal microstructure and has enabled high-resolution quantification of RNFL thickness. OCT has been successfully used to evaluate axonal injury and disease progression in a number of neurological conditions.^2^ In epilepsy, we previously reported a strong linear relationship between RNFL thickness and visual field size in people with a history of vigabatrin exposure.^3^ Only one other study has explored RNFL and macular thickness in epilepsy, in adolescents with newly diagnosed epilepsy before and during monotherapy with either valproic acid or carbamazepine over 1 year duration: no difference was detected.^4^ Widespread white matter involvement has already been shown in epileptogenesis,^5^ and in seizure-related degeneration associated with progressive cognitive decline in epilepsy.^6^ Diffusion tensor imaging and volumetric MRI have demonstrated abnormalities in white matter integrity in people with temporal lobe epilepsy.^7–9^ Widespread neuronal degeneration is also seen in chronic human epilepsy,^10^ and in most animal models of focal epileptogenesis in the immature and adult brain,^11^ suggesting the existence of a disordered ‘connectome’.^9^ ^12^ We postulated that there are common biological mechanisms leading to both neuronal degeneration and RNFL changes in people with epilepsy. We therefore hypothesised that RNFL thinning would occur in epilepsy, independent of previous vigabatrin treatment, and would be associated with clinical and neuroradiological features of epilepsy.

## Methods

### Participants

Four hundred and fifty-four people with epilepsy able to undertake OCT were consecutively included in this cross-sectional cohort study. People were recruited from tertiary care clinics at the National Hospital for Neurology and Neurosurgery from September 2008 to August 2013. Demographic and clinical data were obtained from medical records. Participants were excluded from the analyses if they had previous exposure to vigabatrin, diabetes, glaucoma or other known ocular disease, concurrent diagnosis of multiple sclerosis, history of trauma or surgery to the eye or orbit, a distance refractive error of >4.50 dioptres mean sphere/>2.5 dioptres cylinder, brain MRI evidence of visual pathway involvement (defined as damage to the optic nerve, chiasm or tract, to the lateral geniculate nucleus, to the optic radiations or to the primary or association visual cortices, of traumatic, vascular or inflammatory origin as determined by clinical neuroradiological review). Ninety healthy control participants were recruited from May 2010 to August 2013; the controls also met relevant inclusion and exclusion criteria.

### Clinical data

The following variables were evaluated: age, sex, ethnicity, epilepsy diagnosis,^13^ duration of epilepsy (from the time of diagnosis to the date of OCT assessment), handedness, intellectual disability, antiepileptic drug (AED) history, epilepsy surgery, presence of VNS (vagus nerve stimulator, active or switched off). Intellectual disability was defined as an IQ<70 from a previous psychometric assessment, with onset under 18 years of age, or systematic mention (on at least two occasions) of ‘learning/intellectual disability’ or ‘mental retardation’ in the medical notes. Drug resistance was assessed at the time of OCT assessment and defined as previous failure of adequate trials of two tolerated, appropriately chosen and used AED schedules (whether as monotherapies or in combination) to achieve sustained (>12 months) seizure freedom.^14^ Anyone who had had therapeutic neurosurgical resection for the treatment of epilepsy was considered drug-resistant irrespective of outcome, in keeping with convention. People who did not meet criteria for being classed as drug-resistant were labelled as ‘not-resistant’. We also considered the total number of AEDs to which each person was currently, and ever had been, exposed.

### Optical coherence tomography

**Figure 1:**
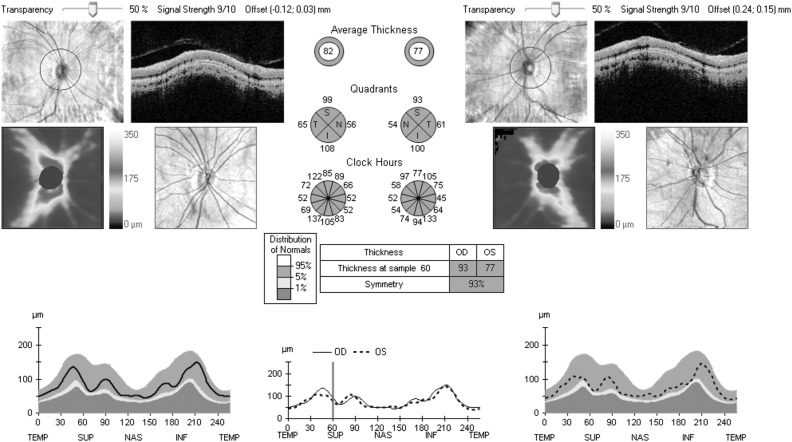
Sample optical coherence tomography report of retinal nerve fibre layer (RNFL) thickness taken from a healthy control with normal visual fields showing normal RNFL thickness with an average RNFL thickness of 82 µm in the right eye and 77 µm in the left eye. The average RNFL thickness and RNFL thickness in each of the 90° quadrants are shown in green as they fall within the ≤95th to ≥5th centile of the normal distribution percentiles provided by the manufacturer's inbuilt database.

All participants were assessed using spectral-domain OCT (Cirrus OCT; Carl Zeiss Meditec, USA), by six experienced operators. Intraobserver and interobserver reproducibility were excellent (see online supplementary data S1–S2). Average RNFL thickness in the peripapillary area and the four 90° quadrants (temporal, nasal, superior and inferior) was measured. The automated report gave the percentile into which the RNFL thickness fell, based on the manufacturer's internal database of age-corrected normal values.^15^ The scan was considered *normal* if the values obtained were between the 5th and 95th centiles, borderline if between 1st and 5th, and abnormally thinned if <1st centile (figure 1). Only data from the right eye were analysed (see online supplementary data S3).

### MRI

Brain MRIs performed within a 12-month interval before or after OCT were evaluated. This time span was a pragmatic choice given previous evidence of an average of 0.92% total brain volume loss, 0.80% grey matter volume loss and 1.11% white matter volume loss over a 3.5 year period in 179 people with epilepsy versus an average of 0.49% total brain volume loss, 0.31% grey matter volume loss and 0.71% white matter volume loss over the same follow-up period in 90 healthy controls.^16^ MRI acquisition and analysis are described in online supplementary data S4.

### Statistics

Data were analysed using Stata/IC V.11.1 (Stata, Texas, USA). t test and Pearson χ^2^ test were used to compare demographic data between cases and controls. Comparison of the distribution of average RNFL thickness, considered both as a continuous variable and as a categorical variable, between cases and controls, was performed by t test and Pearson χ^2^, with a Bonferroni-adjusted p<0.05 significance level. Thereafter, only the group of people with epilepsy was analysed. t test, analysis of variance (ANOVA) and Pearson's correlation coefficient were used to test for differences in the distribution of average RNFL thickness as a continuous variable according to each demographic and clinical factor, considered independently. To identify independent predictors of RNFL changes, the average RNFL thickness was considered as the dependent variable in a multivariate linear regression model, using a backward-stepwise approach. The multivariate regression model was constructed after adjusting for potential confounding factors, considered as the variables that emerged as significant (p<0.05) in the univariate analyses. To identify significant independent predictors of borderline attenuation or abnormal thinning of the RNFL, a multivariate logistic regression model, using a backward-stepwise approach and considering the presence of RNFL thinning below 5th centile as the dependent variable was generated. We estimated ORs and 95% CIs. Variables with high collinearity (variance inflation factor >5) were excluded from the multivariate model. The set of people with available MRI was divided into two groups according to scan state (normal or abnormal—see online supplementary table S1). Analysis of correlation between average RNFL thickness, sex, duration of epilepsy and neuroimaging volumetric data was first performed in the two subgroups and then in the entire subset (Pearson's correlation, Bonferroni correction for sex and duration).

### Standard protocol approvals, registrations and consent

This study was approved by the institutional ethics committee. All participants provided written informed consent.

## Results

Three hundred of the 454 people scanned met inclusion criteria (exclusions listed in online supplementary data S5). Their scans were analysed and compared to results from 90 healthy controls. Most people had drug-resistant epilepsy (210 individuals, 72.2%) and long disease duration (mean 23.3 years, SD 15.1). Demographic and clinical data are presented in online supplementary table S2.

### RNFL thickness in people with epilepsy compared with healthy controls

The average RNFL thickness and the thickness of each 90° quadrant were significantly lower in people with epilepsy than in healthy controls (table 1): 12.7% had abnormal average RNFL thinning (≤1st centile), with 17.0% having borderline changes (≤5th to >1st centile). RNFL thinning ≤1st centile, or >1st to 5th centile, was most frequently seen in the superior and inferior quadrants (table 2). As expected, ≥95% of healthy controls showed normal average and 90° quadrant RNFL thickness (>5th centile). No control had abnormal RNFL thinning (<1st centile) for average RNFL thickness or for any 90° quadrant.

**Table 1:** RNFL thickness, considered as continuous variable, in people with epilepsy and in healthy controls

Average RNFL thickness, µm	People with epilepsy	Healthy controls	Significance of difference (t test), p Value
Average RNFL thickness across all 4 quadrants, mean±SD	85.4±14.3	95.3±8.8	<0.001
Superior quadrant	103.4±22.4	115.3±10.6	<0.001
Nasal quadrant	68.9±14.7	77.7±13.9	<0.001
Inferior quadrant	107.8±24.1	122.8±14.5	<0.001
Temporal quadrant	61.4±13.6	65.2±10.2	0.016

RNFL, retinal nerve fibre layer.

**Table 2:** RNFL thickness, considered as categorical variable according to the normal distribution percentiles provided by the manufacturer's inbuilt database, in people with epilepsy and in healthy controls

	People with epilepsy			Healthy controls			Significance of difference (Pearson χ^2^), p Value*
Average RNFL thickness, µm	N (%)	B (%)	A (%)	N (%)	B (%)	A (%)	
Average RNFL thickness across all 4 quadrants	70.3	17.0	12.7	95.5	4.5	0.0	<0.001
Superior quadrant	71.6	9.3	19.1	100	0.0	0.0	<0.001
Nasal quadrant	89.9	5.4	4.7	97.7	2.3	0.0	0.018
Inferior quadrant	71.6	12.4	16.0	98.9	1.1	0.0	<0.001
Temporal quadrant	88.6	6.0	5.4	96.6	3.4	0.0	0.023

*Comparison of normal versus borderline or abnormal values among the two groups (2×2 table).

A, abnormal (≤1st centile); B, borderline (≤5th to >1st centile); RNFL, retinal nerve fibre layer; N, normal (>5th centile).

### Average RNFL thickness and clinical characteristics—univariate associations

We found a correlation between smaller average RNFL thickness and increasing age (Pearson's correlation coefficient −0.18; p<0.001) and epilepsy duration (Pearson's correlation coefficient −0.14; p=0.015), with both age and duration directly correlated with each other (Pearson's correlation coefficient 0.62; p<0.001). Age was not correlated with RNFL thickness in the modest number of healthy controls (Pearson's correlation coefficient 0.01; p=0.919). Average RNFL thickness was greater in females (88.8 µm±14.0 SD) than males (85.5 µm±13.3 SD; p=0.021, t test). People with epilepsy and intellectual disability had thinner average RNFL (83 µm±12.5 SD) than those with normal intellectual function (86.1 µm±14.8 SD; p=0.049, t test). There was no difference in the distribution of RNFL thickness according to handedness (p=0.523, ANOVA), epilepsy type (p=0.219, ANOVA) or ethnicity (p=0.436, ANOVA).

### Average RNFL thickness across all quadrants and exposure to AEDs or non-medical treatments

Average RNFL thinning was associated with exposure to the following individual AEDs (correlation analysis, uncorrected p values): ethosuximide, phenytoin, primidone, sodium valproate and topiramate. The effect of different AED combinations on RNFL thickness was not tested. The presence of VNS (either active or switched off) was also associated with thinner average RNFL thickness. Previous surgical treatment for epilepsy (n=31) was not associated with a significantly thinner RNFL (see online supplementary table S3). However, operated patients had thinner average RNFL (82.5 µm±15.7 SD) than healthy controls (95.3 µm±8.8 SD; p<0.001, t test). The interval between the surgical treatment to OCT scan was not significantly correlated with the RNFL thickness in this small sample of 31 operated cases (Pearson's correlation coefficient −0.09; p=0.647). There was no significant correlation between the average RNFL thickness and the total number of AEDs taken (Pearson's correlation coefficient −0.09; p=0.154). People with drug-resistant epilepsy had thinner average RNFL (84.2 µm±13.9 SD) than people with non-resistant epilepsy (88.1 µm±15.3 SD; p=0.038, t test), who in turn had thinner average RNFL than healthy controls (95.3 µm±8.8 SD; p=0.001, t test). A significant association between drug resistance and VNS implant (p=0.004, Pearson χ^2^) was found. In the drug-resistant group, there were more females (54.8%) than males (45.2%; p=0.042, Pearson χ^2^).

### Predictors of RNFL thickness changes in multivariate regression models

Univariate linear regression analysis showed significant association between RNFL and: female sex, epilepsy duration, intellectual disability, exposure to ethosuximide, phenytoin, primidone, valproate or topiramate, VNS implant, and drug-resistant epilepsy. Multivariate linear regression showed significant association for RNFL thinning only with epilepsy duration, intellectual disability, drug resistance (adjusted R^2^=0.072, p<0.001; table 3) The residuals of the model were close to a normal distribution (0.37% low severe outliers, 0.00% high severe outliers; mean variance inflation factor 1.03).

**Table 3:** Univariate and multivariate linear regression analysis considering average RNFL thickness across all quadrants as dependent variable

Variables	Univariate coefficient (95% CI)	Multivariate coefficient	VIF	t	p Value	95% CI
Epilepsy duration	−0.16 (−0.27 to −0.06)	−0.17	1.04	−3.01	0.003	−0.27 to −0.06
Intellectual disability	−4.49 (−8.38 to −0.59)	−3.95	1.03	−2.02	0.048	−7.87 to −0.04
Drug resistance	−6.07 (−9.69 to −2.46)	−4.89	1.01	−2.65	0.009	−8.52 to −1.25
Female sex	4.19 (0.98 to 7.41)	–	–	–	–	–
Ethosuximide exposure	−7.07 (−13.23 to −0.90)	–	–	–	–	–
Phenytoin exposure	−4.58 (−8.00 to −1.16)	–	–	–	–	–
Primidone exposure	−8.68 (−15.66 to −1.69)	–	–	–	–	–
Sodium valproate exposure	−4.25 (−7.97 to −0.53)					
Topiramate exposure	−4.45 (−7.74 to −1.17)					
VNS implant	−11.07 (−17.64 to −4.51)					
Constant		94.14	1.94	48.52	<0.001	90.32 to 97.96

RNFL, retinal nerve fibre layer; VIF, variance inflation factor; VNS, vagus nerve stimulator.

Multivariate logistic regression analysis showed an increased probability of RNFL thinning (borderline or abnormal) in people with drug-resistant epilepsy (OR=2.09, CI 95% 1.09 to 4.01, p=0.027). A global test of goodness-of-fit indicated that the model fitted the data well (Hosmer and Lemeshow’s goodness-of-fit test, Pearson χ²=175.45, p=0.497). Neither multivariate linear nor logistic models that considered the total number of AEDs in place of drug resistance showed an association.

### Correlation between average RNFL thickness and MRI brain volumes

Of 300 people with epilepsy, 211 had an MRI performed within±12 months of OCT (median 0 days; range −304 to +350). Thirty-nine scans were excluded due to poor segmentation or lack of a T1-weighted volumetric (three-dimensional) sequence; 172 MRIs were included (107 interpreted as normal, 65 as abnormal, listed in online supplementary table S1). Results are given in online supplementary table S4. In 107 people with epilepsy and normal MRI, we found a significant association between RNFL thickness and brain parenchymal fraction, independent of sex and duration of epilepsy (Pearson's correlation, p=0.010; see online supplementary figure S1). This association was also observed in the group of people with epilepsy and abnormal MRI, after correcting for sex and duration of epilepsy (Pearson's correlation, p=0.045). There was also an overall association between average RNFL thickness and grey matter fraction (Pearson's correlation, p=0.022), which was not seen in the two subgroups with normal (Pearson's correlation, p=0.175) and abnormal (Pearson's correlation, p=0.545) MRI.

## Discussion

We demonstrate that individuals with epilepsy on treatment, but not exposed to vigabatrin, have a thinner RNFL thickness than healthy participants. RNFL thinning is associated with longer duration of epilepsy, presence of drug resistance and intellectual disability. In the subset of people with epilepsy and available MRI data, brain parenchymal fraction shows a direct linear association with the average RNFL thickness. Only drug resistance emerges as a significant independent predictor of borderline attenuation or abnormal thinning of the RNFL in a logistic regression model. People with drug-resistant epilepsy have a more than twofold odds of RNFL thinning compared with people with non-resistant epilepsy.

The biological mechanisms leading to the association between RNFL changes and intellectual disability, lower brain parenchymal fraction or drug resistance are unknown. Retina and brain share common embryonic origin and patterns of gene expression.^17^ A physical association between these two structures of neuroectodermal origin is also manifest through trans-synaptic degeneration, a process occurring when damage spreads from posterior to anterior visual pathway or vice versa, described in several central nervous system diseases, with neuronal damage caused by a focal brain lesion affecting the function and morphology of remote, apparently intact, regions following interruption of brain circuits.^18^ In people with temporal lobe epilepsy, trans-synaptic degeneration of the limbic system or extratemporal areas has been demonstrated using MRI, positron emission tomography and single-photon emission CT imaging.^19^ Widespread white and grey matter involvement has already been shown in epilepsy.^10–11^ ^20^ Evidence of trans-synaptic degeneration in the human central nervous system has already been shown in previous studies using OCT.^21^ ^22^ These links may underpin the observed associations between RNFL thinning and various measures, structural (eg, brain parenchymal fraction) and functional (intellectual disability, drug resistance).

Several previous studies have suggested that RNFL thickness reflects cerebral axonal integrity.^23^ ^24^ Given the association between RNFL thickness and brain parenchymal fraction (brain parenchymal volume normalised for baseline differences among participants)^25^ in multiple sclerosis,^23^ independent of optic neuritis,^24^ RNFL thickness has been proposed as a marker of early neurodegenerative processes in multiple sclerosis. Shared neurodegenerative processes have also been demonstrated in a mouse model of Alzheimer's disease, where β-amyloid immunoreactive plaques were detected in the retina.^26^ RNFL thinning has been shown in a wide spectrum of neurological and ophthalmological conditions, with a highly variable interval from an acute event or disease onset to thinning, ranging from months after an acute attack of acute angle closure glaucoma^27^ or optic neuritis^28^ to years as, for instance, in multiple sclerosis.^29^ Shared neurodegenerative processes may also be present in the epilepsies. We report, for the first time in epilepsy, a significant association between RNFL thinning and lower brain volume as assessed by brain MRI. This association was maintained after correcting for sex and duration of epilepsy. From our cross-sectional study, we cannot determine the reasons for lower brain parenchymal fraction, but RNFL thickness does seem to reflect this cerebral measure. A cross-sectional relationship may not yield the strongest correlations since brain atrophy may lag behind RNFL changes or vice versa. If this association was confirmed in future prospective studies, RNFL thickness could be advanced as a reliable, inexpensive and easily assessed complementary surrogate marker for exploration of whole-brain cerebral processes, such as potential neurodegenerative processes,^11^ in human epilepsy.

Seizures have a number of adverse consequences.^30^ Drug resistance is the label applied when seizures continue to occur despite treatment. In our analysis, drug resistance is the only independent predictor of a borderline or abnormal RNFL thickness. One possible explanation is that ongoing seizures cause cerebral damage that may manifest in lower brain parenchymal fraction and secondary thinning of RNFL. Longitudinal studies are now needed to verify this. There are alternative interpretations, such as the concept of ‘intrinsic epilepsy severity’, which holds that there are common neurobiological factors underlying both severity and drug resistance in epilepsy.^31^ Developing this theory, thinner RNFL, smaller brain parenchymal fraction and presence of intellectual disability might all be part of a more severe epilepsy condition from outset. Additional effects over time may further aggravate RNFL thinning. Prospective studies will be needed to disentangle these various possibilities.

Other associations with RNFL thinning were found: epilepsy duration, intellectual disability, VNS implant. Epilepsy duration showed a significant linear inverse relationship with RNFL thickness, though it did not predict abnormal or borderline RNFL thinning in the logistic multivariate model. Age at examination was also associated with RNFL thinning in the independent association analysis, but was excluded from the regression models because of high collinearity with epilepsy duration. A thinner average RNFL was found in people with epilepsy and intellectual disability than in people with epilepsy and normal intellectual function. The underlying cause of intellectual difficulties might also influence drug responsiveness. In our analysis, intellectual disability as a variable was extracted from medical record review and in most cases was confirmed by formal neuropsychometry. Intellectual disability present in children born prematurely has been attributed to injury to the cerebral white matter and associated neuronal and axonal abnormalities.^32^ However, when considering RNFL thickness in children, its association with the development of the anterior chamber needs to be taken in account and a number of potential confounding factors have to be considered, including age, axial eyeball length and refractive status.^33^ In studies of individuals with multiple sclerosis, RNFL thinning was associated with cognitive disability.^34^ Similarly, in healthy young individuals, RNFL thickness was associated with level of cognitive functioning.^35^ The association of RNFL thinning with intellectual disability or cognitive impairment may therefore also reflect compromised white matter integrity.^36^ ^37^ VNS implant was significantly related with RNFL thinning in the univariate linear regression model, probably due to high collinearity with drug-resistant epilepsy. Thinning seems unrelated to syndromic diagnosis, but this will need to be explored in a larger study testing RNFL thinning in specific epilepsy subtypes.

There are limitations to our study. A key caveat is the cross-sectional design, which cannot ascribe causation. Prospective studies are needed to evaluate the role of RNFL thickness as a possible biomarker and to tease out the effects of other factors, such as the frequency of seizures and the use of specific AEDs, some of which have been reported to influence brain volume or resilience to injury. We recognise that, using retrospective case note data not designed for such studies, the dichotomous classification into ‘drug-resistant’ and ‘non-resistant’ is an oversimplification in absolute terms, but we note that the total number of drugs tried by the two groups is significantly different in the expected direction, so that any oversimplification is unlikely to have a material impact on our findings. The study included fewer controls than cases, though cases and controls were well matched in terms of age, sex and ethnicity, which are the only non-pathological factors known to influence RNFL thickness. All data were also compared with a larger internal data set from normal individuals. We note also that while the total number of people studied was 300, this number included people with all types of epilepsy. Some syndromes may have a greater propensity for neurodegeneration and white matter volume loss, and possibly therefore also for RNFL thinning. Though we excluded participants with known glaucoma, we recognise that glaucoma might be asymptomatic or undiagnosed. Furthermore, individuals with intraocular pressure in the normal range might still have normal-tension glaucoma and RNFL thinning. All these issues will need further exploration in a larger cohort followed prospectively.

OCT is a fast, reproducible, non-invasive, well-tolerated and cost-efficient investigation. When compared with other potential methods for investigating resistance,^16^ ^38^ ^39^ OCT is not subject to temporal sampling issues like EEG, represents about 5% of the capital outlay of typical MRI, is much cheaper to run and can be undertaken and interpreted by easily trained operators. If confirmed in a prospective study, our findings might have clinical relevance. RNFL thickness might be considered as an objective, complementary and repeatable measure of the biology of drug resistance, and possibly disease severity. Furthermore, our results provide a basis for a better understanding of mechanisms underlying neurodegeneration in epilepsy.

## Supplementary Material

Web supplement

Web figure

## References

[1] Frohman E, Costello F, Zivadinov R, et al Optical coherence tomography in multiple sclerosis. Lancet Neurol 2006;5:853–63. 10.1016/S1474-4422(06)70573-7 16987732

[2] Jindahra P, Hedges TR, Mendoza-Santiesteban CE, et al Optical coherence tomography of the retina: applications in neurology. Curr Opin Neurol 2010;23:16–23. 10.1097/WCO.0b013e328334e99b 20009925

[3] Clayton LM, Dévilé M, Punte T, et al Retinal nerve fibre layer thickness in vigabatrin-exposed patients. Ann Neurol 2011;69:845–54. 10.1002/ana.22266 21246602

[4] Lobefalo L, Rapinese M, Altobelli E, et al Retinal nerve fiber layer and macular thickness in adolescents with epilepsy treated with valproate and carbamazepine. Epilepsia 2006;47:717–19. 10.1111/j.1528-1167.2006.00505.x 16650137

[5] Buzsaki G, Ponomareff GL, Bayardo F, et al Neuronal activity in the subcortically denervated hippocampus: a chronic model for epilepsy. Neuroscience 1989;28:527–38. 10.1016/0306-4522(89)90002-X 2710328

[6] Hermann B, Seidenberg M, Bell B, et al The neurodevelopmental impact of childhood-onset temporal lobe epilepsy on brain structure and function. Epilepsia 2002;43:1062–71. 10.1046/j.1528-1157.2002.49901.x 12199732

[7] Liu RS, Lemieux L, Bell GS, et al Progressive neocortical damage in epilepsy. Ann Neurol 2003;53:312–24. 10.1002/ana.10463 12601699

[8] Sisodiya SM, Moran N, Free SL, et al Correlation of widespread preoperative magnetic resonance imaging changes with unsuccessful surgery for hippocampal sclerosis. Ann Neurol 1997;41:490–6. 10.1002/ana.410410412 9124806

[9] Behrens TE, Sporns O Human connectomics. Curr Opin Neurobiol 2012;22:144–53. 10.1016/j.conb.2011.08.005 21908183 PMC3294015

[10] Thom M, Eriksson S, Martinian L, et al Temporal lobe sclerosis associated with hippocampal sclerosis in temporal lobe epilepsy: neuropathological features. J Neuropathol Exp Neurol 2009;68:928–38. 10.1097/NEN.0b013e3181b05d67 19606061 PMC2723771

[11] Holopainen IE Seizures in the developing brain: cellular and molecular mechanisms of neuronal damage, neurogenesis and cellular reorganization. Neurochem Int 2008;52:935–47. 10.1016/j.neuint.2007.10.021 18093696

[12] Bernasconi N, Duchesne S, Janke A, et al Whole-brain voxel-based statistical analysis of gray matter and white matter in temporal lobe epilepsy. Neuroimage 2004;23:717–23. 10.1016/j.neuroimage.2004.06.015 15488421

[13] [No authors listed] Proposal for revised classification of epilepsies and epileptic syndromes. Commission on Classification and Terminology of the International League Against Epilepsy. Epilepsia 1989;30:389–99. 10.1111/j.1528-1157.1989.tb05316.x 2502382

[14] Kwan P, Arzimanoglou A, Berg AT, et al Definition of drug resistant epilepsy: consensus proposal by the ad hoc Task Force of the ILAE Commission on Therapeutic Strategies. Epilepsia 2010;51:1069–77. 10.1111/j.1528-1167.2009.02397.x 19889013

[15] Knight OJ, Girkin CA, Budenz DL, et al, Cirrus OCT Normative Database Study Group. Effect of race, age, and axial length on optic nerve head parameters and retinal nerve fibre layer thickness measured by Cirrus HD-OCT. Arch Ophthalmol 2012;130:312–18. 10.1001/archopthalmol.2011.1576 22411660 PMC5536837

[16] Liu RS, Lemieux L, Bell GS, et al Cerebral damage in epilepsy: a population-based longitudinal quantitative MRI study. Epilepsia 2005;46:1482–94. 10.1111/j.1528-1167.2005.51603.x 16146444

[17] Hackam AS, Qian J, Liu D, et al Comparative gene expression analysis of murine retina and brain. Mol Vis 2004;10:637–49. 15359217

[18] Matthews MA Death of the central neuron: an electron microscopic study of thalamic retrograde degeneration following cortical ablation. J Neurocytol 1973;2:265–88. 10.1007/BF01104030 9224491

[19] Takaya S, Mikuni N, Mitsueda T, et al Improved cerebral function in mesial temporal lobe epilepsy after subtemporal amygdalohippocampectomy. Brain 2009;132:185–94. 10.1093/brain/awn218 18790818

[20] Concha L, Beaulieu C, Collins DL, et al White-matter diffusion abnormalities in temporal-lobe epilepsy with and without mesial temporal sclerosis. J Neurol Neurosurg Psychiatry 2009;80:312–19. 10.1136/jnnp.2007.139287 18977826

[21] Mehta JS, Plant GT Optical coherence tomography (OCT) findings in congenital/long-standing homonymous hemianopia. Am J Ophthalmol 2005;140:727–9. 10.1016/j.ajo.2005.03.059 16226527

[22] Jindahra P, Petrie A, Plant GT The time course of retrograde trans-synaptic degeneration following occipital lobe damage in humans. Brain 2012;135:534–41. 10.1093/brain/awr324 22300877

[23] Gordon-Lipkin E, Chodkowski B, Reich DS, et al Retinal nerve fibre layer is associated with brain atrophy in multiple sclerosis. Neurology 2007;69:1603–9. 10.1212/01.wnl.0000295995.46586.ae 17938370

[24] Dörr J, Wernecke KD, Bock M, et al Association of retinal and macular damage with brain atrophy in multiple sclerosis. PLoS ONE 2011;6:e18132 10.1371/journal.pone.0018132 21494659 PMC3072966

[25] Phillips MD, Grossman RI, Miki Y, et al Comparison of T2 lesion volume and magnetization transfer ratio histogram analysis and of atrophy and measures of lesion burden in patients with multiple sclerosis. AJNR Am J Neuroradiol 1998;19:1055–60. 9672011 PMC8338648

[26] Liu B, Rasool S, Yang Z, et al Amyloid-peptide vaccinations reduce {beta}-amyloid plaques but exacerbate vascular deposition and inflammation in the retina of Alzheimer's transgenic mice. Am J Pathol 2009;175:2099–110. 10.2353/ajpath.2009.090159 19834067 PMC2774073

[27] Lee JW, Woo TT, Yau GS, et al Cross-sectional study of the retinal nerve fiber layer thickness at 7years after an acute episode of unilateral primary acute angle closure. Medicine (Baltimore) 2015;94:e391 10.1097/MD.0000000000000391 25590844 PMC4602553

[28] Yau GS, Lee JW, Lau PP, et al Prospective study on retinal nerve fibre layer thickness changes in isolated unilateral retrobulbar optic neuritis. Sci World J 2013;2013:694613. 10.1155/2013/694613 PMC3886364 24459442

[29] Abalo-Lojo JM, Limeres CC, Gómez MA, et al Retinal nerve fiber layer thickness, brain atrophy, and disability in multiple sclerosis patients. J Neuroophthalmol 2014;34:23–8. 10.1097/WNO.0000000000000057 24162258

[30] Novy J, Belluzzo M, Caboclo LO, et al The lifelong course of chronic epilepsy: the Chalfont experience. Brain 2013;136:3187–99. 10.1093/brain/awt117 23824485

[31] Rogawski MA, Johnson MR Intrinsic severity as a determinant of antiepileptogenic drug refractoriness. Epilepsy Curr 2008;8:127–30. 10.1111/j.1535-7511.2008.00272.x 18852835 PMC2566613

[32] Volpe JJ Brain injury in premature infants: a complex amalgam of destructive and developmental disturbances. Lancet Neurol 2009;8:110–24. 10.1016/S1474-4422(08)70294-1 19081519 PMC2707149

[33] Lee JW, Yau GS, Woo TT, et al The anterior chamber depth and retinal nerve fiber layer thickness in children. Sci World J 2014;2014:538283 10.1155/2014/538283 PMC4241318 25431789

[34] Toledo J, Sepulcre J, Salinas-Alaman A, et al Retinal nerve fiber layer atrophy is associated with physical and cognitive disability in multiple sclerosis. Mult Scler 2008;14:906–12. 10.1177/1352458508090221 18573835

[35] van Koolwijk LM, Despriet DD, Van Duijn CM, et al Association of cognitive functioning with retinal nerve fiber layer thickness. Invest Ophthalmol Vis Sci 2009;50:4576–80. 10.1167/iovs.08-3181 19420335

[36] Hermann B, Seidenberg M, Bell B, et al Extratemporal quantitative MR volumetrics and neuropsychological status in temporal lobe epilepsy. J Int Neuropsychol Soc 2003;9:353–62. 12666760 10.1017/S1355617703930013

[37] McDonald CR, Ahmadi ME, Hagler DJ, et al Diffusion tensor imaging correlates of memory and language impairments in temporal lobe epilepsy. Neurology 2008;71:1869–76. 10.1212/01.wnl.0000327824.05348.3b 18946001 PMC2676974

[38] Bonnett L, Smith CT, Smith D, et al Prognostic factors for time to treatment failure and time to 12months of remission for patients with focal epilepsy: post-hoc, subgroup analyses of data from the SANAD trial. Lancet Neurol 2012;11:331–40. 10.1016/S1474-4422(12)70018-2 22377180

[39] Feldmann M, Asselin MC, Liu J, et al P-glycoprotein expression and function in patients with temporal lobe epilepsy: a case-control study. Lancet Neurol 2013;12:777–85. 10.1016/S1474-4422(13)70109-1 23786896

