# Supplementary material for: Retinal nerve fibre layer thinning is associated with drug resistance in epilepsy
Source: J Neurol Neurosurg Psychiatry. 2015 Apr 17;87(4):396–401. doi: 10.1136/jnnp-2015-310521 (PMC4819648; doi:10.1136/jnnp-2015-310521)
Supplement: Web supplement [file jnnp-2015-310521-s1.pdf]

S1- OCT reliability

Others publications have reported satisfactory intra- and inter-observer reproducibility of Cirrus OCT measuring RNFL thickness in normal eyes with refractive errors <5 dioptres of spherical equivalent refraction or 3 dioptres of astigmatism. Indeed, they showed clinically-acceptable repeatability and reproducibility of the technique, with coefficients of variation below 5%[1].Another recent study found almost perfect agreement between examiners with Cirrus OCT in the global RNFL, inferior, and superior quadrants, and at the 5, 6, and 12 o'clock positions; they also showed that the signal strength is independent of the examiner's experience in Cirrus OCT[2]. We performed an intra- and inter-examiner reliability assessment; our inter-examiner reliability was classified as excellent according to commonly-employed criteria[3].

Assessment of intra-operator reliability

The paired measures were obtained by the same operator (SB) assessing the same patient in the same session, considering two different quality thresholds (threshold 1 from 7 to 10; threshold 2= threshold 1 ±1), in 63 people with epilepsy. Intra-observer agreement and repeatability analyses of RNFL showed that there was no significant difference between the two measurements.

Bland-Altman comparison of RNFL measure 1 and RNFL measure 2:

Limits of agreement (Reference Range for difference): -17.8 µm to 19.0 µm

Mean difference: 0.57 µm (CI -1.75 to 2.89)

Range: 38.0 µm to 169.0 µm

Pitman's Test of difference in variance: r = -0.018, n = 63, p = 0.89

Pitman's variance ratio test between first average RNFL measure and second average RNFL measure:

Ratio of Standard deviations = 0.99

95% Confidence Interval 0.90 to 1.10

t = -0.141, df = 61, p = 0.89

Assessment of inter-operator reliability for RNFL measures (4 different operators) Intraclass correlation coefficient (ICC) for RNFL measures was estimated by multi-level regression. We obtained an ICC of 0.830 [95% CI 0.713-0.911].

Assessment of inter-operator reliability for quality strength values (4 different operators)

Intraclass correlation coefficient (ICC) for quality strength values was estimated by multi-level regression. We obtained an ICC of 0.752 [95% CI 0.599-0.867].

S2-OCT Methodology

The peri-papillary RNFL thickness was measured using the Optic Disc Cube 200x200 protocol. A 6x6mm grid of data is acquired with 200 horizontal scans, each composed of 200 A-scans that are centered over the optic disk. The participant was positioned at the machine and asked to fixate on an internal target. When necessary, polarization was optimized with the automatic tool or manually. Once the optic disc was clearly visualised, the scan circle was centered on the optic disk. The position of the scan circle was analysed according to its relation to the optic nerve, and repositioned if necessary. We attempted to obtain at least three scans of both eyes, always starting with the right. Scans were analysed according to quality, visualisation of optic nerve, movement artefacts and position of scan circle. The software provides a quality rating between 0-10 arbitrary units; scans were excluded if quality fell below 7. Quality measure variation above this threshold of 7 did not influence RNFL thickness measurement. The right eye scan with highest signal strength was selected for analysis for each individual. If more than one scan had equal signal strength, the last scan was selected.

S3- "One-eye design"

A "one-eye design"[4] was chosen for this study so that standard statistical techniques could be applied to analyse the data without increasing the risk of Type 1 errors. Analyses in which both eyes of an individual are used ("two-eye designs") require particular statistical approaches. This is because, within an individual, data from one eye are more likely to be similar to data from the other eye of the same individual, than it is from another eye from a different individual, i.e. within a subject the data from each eye will be highly correlated (unless there is unilateral eye disease)[4-5]. This is due to multiple factors including genetic and environmental effects that will act within an individual to influence any structural or functional parameters[5]. In standard statistical analysis, data should be independent, and failure to take account of between-eye correlation in a "two-eye design" can lead to an overestimation of the precision of the statistical values[4-5]. When a "two-eye design" is used for the analysis data from both eyes should be averaged and the average value can be treated with the same standard statistical techniques as a "single-eye design". Alternatively, more complex statistical approaches are needed to determine the contribution to the outcome measure of variance arising from between-subject factors and those arising from within-subject factors[4]. A further complicating factor is added to both of these "two-eye design" approaches when some individuals only have data from one eye. In our study, whilst many individuals had data from both eyes, some individuals only had data from only one eye.

For a "one-eye" design, the eye to be entered into the analysis can either be chosen at random, or can be chosen to be always the right eye, or always the left eye[5]. Recently, this observation has also been made in a group of 248 healthy volunteers, where the average RNFL thickness in the right eye was 0.52µm thicker than the average RNFL thickness in the left eye, which reached statistical significance[6]. Thus only the right eye was chosen from each individual to use in the analysis.

S4- Neuroimaging methodology

All MRI scans were acquired on a 3 T GE Signa HDx scanner (General Electric, Milwaukee, Wisconsin, U.S.A.). Standard imaging gradients with a maximum strength of 40 mT/m and slew rate 150 T/m/s were used. Clinical sequences performed included a coronal T1-weighted volumetric (3D) acquisition with 170 contiguous 1.1 mm thick slices (matrix 256 × 256, in-plane resolution 0.9375 × 0.9375 mm). Dicom image files were converted into NIfTI format for VBM (voxel-based morphometry) analysis. T1-weighted images were fully automated segmented into grey matter, white matter, and cerebrospinal fluid using Statistical Parametric Mapping (SPM) 8 software (Wellcome Department of Imaging Neuroscience, London, England, www.fil.ion.ucl.ac.uk) and the Voxel Based Morphometry (VBM) 8 toolbox (http://dbm.neuro.uni-jena.de/vbm8/). All segmentations were verified by a trained observer. Poor quality scans with subsequent unsatisfactory segmentation and scans without T1-weighted volumetric (3D) sequence were excluded. Global tissue volumes, including grey matter volume, white matter volume and total intracranial volume, were then estimated for each patient. Total intracranial volume was the sum of total (whole brain) cerebrospinal fluid and grey and white matter volumes. Grey matter fraction and white matter fraction were calculated by dividing the grey and white matter volumes respectively by total intracranial volume. Brain parenchymal fraction was calculated by dividing the summed volume of grey and white matter by total intracranial volume[7].

Table S1. List of available MRI scans within 12 months either side of the OCT assessment, reported by a consultant neuroradiologist as normal or with an abnormality.

Total number of available MRI scans=172	Normal (n=107, 62%)	
	Abnormal (n=65, 38%)	Tumours (n=11, 17%)
		Cerebrovascular lesions and age-related white matter abnormalities (n=14, 22%)
		Malformation of cortical development (n=15, 23%)
		Surgical resection (n=12, 18%)
		Hippocampal sclerosis (n=13, 20%)

S5- Study profile with exclusions

A total of 454 people with epilepsy were consecutively recruited and underwent OCT scan. Of these, 154 people were excluded according to the exclusion criteria (130 had previous vigabatrin exposure documented in medical records, 16 had diabetes, glaucoma or other known ocular disease, 3 had overt brain MRI evidence of occipital or visual pathway involvement, 2 had concurrent diagnosis of multiple sclerosis, 3 OCT scans below the minimum quality criteria or with significant movement artifact). Three hundred people with epilepsy therefore met inclusion criteria and were included in the analysis. Their scans were analysed and compared to results from 90 healthy controls participants who were consecutively recruited and included in the analysis.

Table S2. Characteristics of all participants.

Characteristics		People with epilepsy	Healthy controls	Significance of difference (test)
Age at examination, yrs (mean ± SD)		39.0±13.2	41.9±13.1	p=0.072 (*t*-test)
Sex, number female (%)		175 (58.3)	54 (60.0)	p=0.778 (Pearson χ^2)
Ethnicity, number European (%)		242 (80.7)	69 (76.7)	p=0.408 (Pearson χ2)
Handedness, number (%)	Right	240 (80.0)	NA	
	Left	51 (17.1)		
	Both	5 (1.6)		
	Unknown	4 (1.3)		
Duration of epilepsy, yrs (mean ± SD)		23.3±15.1	NA	-
Type of epilepsy, number (%)	LRE C.	92 (30.7)	NA	-
	LRE S.	117 (39.0)		
	IGE	49 (16.3)		
	UE	42 (14.0)		
Intellectual disability, number (%)		61 (20.7)	NA	-
Drug-resistant, number (%)		210 (72.2)	NA	-

NA= not available/applicable

LRE C.= Localization-Related Cryptogenic, LRE S.= Localization-Related Symptomatic, IGE= Idiopathic Generalised Epilepsy, UE= Unclassified Epilepsy (Commission on Classification and Terminology of the International League Against Epilepsy, 1989)

Table S3. Lifetime AED exposure, other non-medical treatments and association with average RNFL thickness.

Treatment	Number of patients exposed (%)	Average RNFL (µm) in people exposed to the drug	Average RNFL (µm) in people not exposed to the drug	Significance of difference (p-value)*
Acetazolamide	151 (53.7)	86.1	85.0	0.554
Carbamazepine	221 (77.3)	85.3	86.9	0.433
Clobazam	162 (56.6)	85.1	86.8	0.317
Clonazepam	45 (16.1)	85.6	85.6	0.975
Ethosuximide	21 (7.5)	79.3	86.4	0.025
Gabapentin	39 (13.9)	85.9	85.6	0.916
Lacosamide	59 (20.8)	82.9	86.7	0.069
Lamotrigine	206 (71.8)	86.3	84.9	0.438
Levetiracetam	237 (81.2)	85.8	85.9	0.982
Oxcarbazepine	72 (25.4)	85.5	85.9	0.817
Phenobarbital	71 (25.5)	84.8	85.9	0.577
Phenytoin	104 (37.1)	82.9	87.4	0.009
Pregabalin	46 (16.3)	84.3	86.1	0.432
Primidone	16 (5.7)	77.7	86.4	0.015
Sodium valproate	204 (72.3)	84.5	88.7	0.025
Topiramate	135 (47.4)	83.5	87.9	0.008
Zonisamide	65 (23.1)	85.5	85.8	0.858
Surgery	31 (10.9)	82.5	86.5	0.137

VNS	20 (7.9)	75.6	86.7	0.001

*Difference in RNFL thickness between exposed and non-exposed patients (*t*-test) - all uncorrected p-values

Table S4. Relationship between average RNFL thickness and brain volumetric measures in people with normal and lesional MRI scan.

	People with epilepsy and normal MRI (n=107)	People with epilepsy and abnormal MRI (n=65)	Total (n=172)
MRI volumetric measures	Pearson's coefficient values (p-values)		
White matter fraction	0.12 (0.216)<br>0.12 (1.000)[a]	0.14 (0.265)<br>0.14 (1.000)[a]	0.13 (0.103)<br>0.13 (0.621)[a]
Grey matter fraction	0.21 (0.029)<br>0.21 (0.175)	0.21 (0.091)<br>0.21 (0.545)[a]	0.22 (0.004)<br>0.22 (0.022)[a]
Brain parenchymal fraction	0.29 (0.002)<br>0.29 (0.010)[a]	0.33 (0.008)<br>0.33 (0.045)[a]	0.32 (<0.001)<br>0.32 (<0.001)[a]
Total intracranial volume	-0.03 (0.790)<br>-0.03 (1.000)[a]	0.26 (0.039)<br>0.26 (0.235)[a]	0.08 (0.276)<br>0.08 (1.000)[a]

[a] Pearson's coefficient values after correction for sex and duration of epilepsy

Figure S1. Plot showing linear association between average RNFL thickness and brain parenchymal fraction (BPF) in people with normal (a) and abnormal (b) MRI scans.

References

1. Garcia-Martin E, Pinilla I, Idoipe M, Fuertes I, Pueyo V. Intra and interoperator reproducibility of retinal nerve fibre and macular thickness measurements using Cirrus Fourier-domain OCT. Acta Ophthalmol 2011;89:e23-29.

2. Moreno-Montañés J, Olmo N, García N, Alvarez A, García-Granero M. Influence of examiner experience on the reproducibility of retinal nerve fiber thickness values using Cirrus and Stratus OCTs. J Glaucoma 2013;22:243-249.

3. Fleiss JL. The Design and Analysis of Clinical Experiments. New York, NY: Wiley;1986.

4. Ray WA, O'Day DM. Statistical analysis of multi-eye data in ophthalmic research. Invest Ophthalmol Vis Sci 1985;26:1186-1188.

5. Murdoch IE, Morris SS, Cousens SN. People and eyes: statistical approaches in ophthalmology. Br J Ophthalmol 1998;82:971-973.

6. Mwanza JC, Durbin MK, Budenz DL. Interocular Symmetry in Peripapillary Retinal Nerve Fiber Layer Thickness Measured with the Cirrus HD-OCT in Healthy Eyes. Am J Ophthalmol Epub 2011 Jan 13.

7. Juengling FD, Kassubek J. Standardized calculation of brain parenchymal fraction: an approach to objective assessment of cerebral atrophy. AJNR Am J Neuroradiol 2003;24:1492-1493.
