# Supplementary material for: Retinal nerve fibre layer thinning is associated with drug resistance in epilepsy
Source: J Neurol Neurosurg Psychiatry. 2015 Apr 17;87(4):396–401. doi: 10.1136/jnnp-2015-310521 (PMC4819648; doi:10.1136/jnnp-2015-310521)
Supplement: Web figure [file jnnp-2015-310521-s2.pdf]

Figure S1. Plot showing linear association between average RNFL thickness and brain parenchymal fraction (BPF) in people with normal (a) and abnormal (b) MRI scans.

(a)

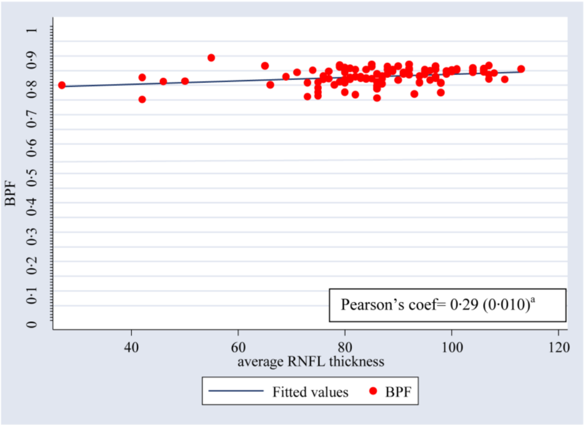

(b)

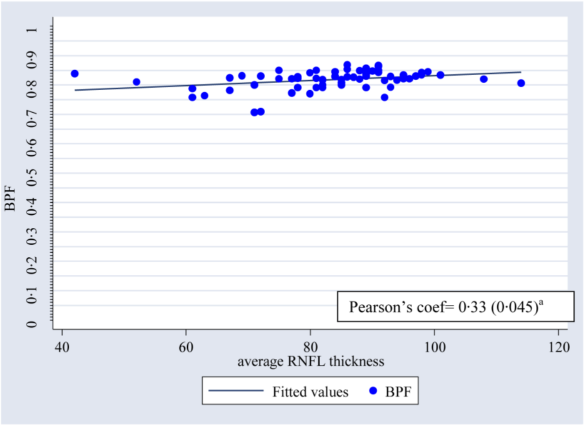

[a]Pearson's coefficient values after correction for sex and duration of epilepsy

BPF= brain parenchymal fraction
